# Cytokine Release Syndrome–Associated Colitis: Insights From a Case of Rituximab-Induced Pancolitis

**DOI:** 10.14309/crj.0000000000001274

**Published:** 2024-02-16

**Authors:** Fnu Vikash, Sindhu Vikash, Michael Mintz, Suzanne Elshafey, Daniel Kim, David Wan

**Affiliations:** 1 Department of Medicine, Albert Einstein College of Medicine/Jacobi Medical Center, Bronx, NY; 2 Department of Gastroenterology, Weill Cornell Medicine, Manhattan, NY

**Keywords:** rituximab, colitis, bright red blood per rectum, cytokine release syndrome, colonoscopy

## Abstract

Rituximab (RTX), a widely used monoclonal antibody for hematologic malignancies and rheumatologic disorders, is known for infusion-related reactions. However, its potential to induce colitis is often overlooked. We present an 85-year-old woman with chronic lymphocytic leukemia experiencing severe adverse effects during her fourth RTX infusion, including abdominal pain, hypotension, and bright red blood per rectum. Computed tomography of the abdomen and pelvis with contrast revealed pancolonic mural wall thickening without perforation. Prompt treatment with vasopressors and intravenous fluids led to symptom resolution within 24 hours. We highlighted the importance of recognizing RTX-induced colitis and discussed and depicted immunological dysregulation mechanisms involved.

## INTRODUCTION

Rituximab (RTX), a monoclonal antibody targeting the CD20 antigen on B lymphocytes, is commonly used for hematologic malignancies and rheumatologic disorders.^1,2^ It selectively targets CD20^+^ B cells on both normal and malignant cells, rapidly depleting B cells through multiple mechanisms and causing immune dysregulation.^3^ RTX is generally well tolerated; however, its widespread use for various medical conditions, including conditions such as refractory idiopathic thrombocytopenic purpura, refractory thrombotic thrombocytopenic purpura, and lymphoproliferative disorders, has led to the identification of rare side effects through postmarketing surveillance.^4,5^ The manufacturer's website outlines potential infusion reactions, but detailed information about the incidence and management of adverse effects is limited.^6^ We present a case of pancolitis after RTX infusion.

## CASE REPORT

An 85-year-old woman with a history of chronic lymphocytic lymphoma (CLL) presented to the emergency department from the infusion clinic because of sudden-onset hypotension, abdominal pain, and bright red blood per rectum (BRBPR) during an RTX infusion. The patient was undergoing her first cycle of RTX at weekly intervals, having received the fourth dose. Before the infusion, she was premedicated with acetaminophen 650 mg, famotidine 20 mg, and loratadine 10 mg. Subsequently, the patient received 600 mg of RTX at the standard infusion rate of 75 mL/hr. Toward the end of the infusion, she experienced intermittent nausea and was administered ondansetron 8 mg.

**Figure 1.**
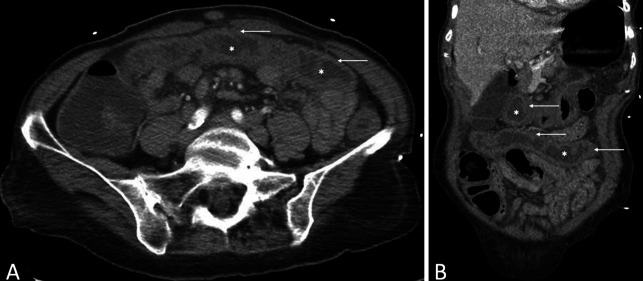
A (axial section) and B (coronal section): gray attenuation pattern indicating pancolitis. Contrast-enhanced computed tomography scans show the distended fluid-filled bowel (asterisks in A and B) and diffuse thickened hypoattenuating bowel wall (arrows in A and B), indicating an inflammatory process without perforation.

After infusion, the patient developed severe abdominal pain and had several episodes of BRBPR. Physical examination revealed diffuse abdominal tenderness, without rebound tenderness or guarding. Bright red blood was noted in the rectal vault during a digital examination. Vital signs included a blood pressure of 50/30 mm Hg, a heart rate of 150 beats per minute, a temperature of 98 °F, and a saturation of 95% on room air. Laboratory results, including complete blood count, comprehensive metabolic panel, and lactate levels, were within normal limits. Contrast-enhanced computed tomography of the abdomen and pelvis revealed pancolonic mural wall thickening without perforation (Figure 1).

Fecal specimens were tested, and results were negative for *Salmonella*, *Shigella*, *Campylobacter*, parasites, as well as *Clostridioides difficile* toxins A and B. Stabilization was achieved through the administration of norepinephrine and fluids, leading to the resolution of signs and symptoms within 24 hours. Because of the rapid improvement observed, colonoscopy was deferred. The patient exhibited no residual gastrointestinal symptoms at discharge and during subsequent follow-ups. To mitigate the risk of further infusion-related reactions, the RTX infusion was continued at a reduced rate.

## DISCUSSION

We present a rare case of RTX-induced pancolitis. To the best of our knowledge, a few published case reports highlight RTX-induced severe colitis necessitating aggressive treatments such as steroids, aminosalicylates, and colectomy.^3,7,8^ Although the median duration of symptoms in reported cases was 21 days,^9^ Our case stands out because of the rapid symptom improvement without additional interventions. To the best of our knowledge, this specific outcome has not been documented in the existing literature.

RTX, a murine immunoglobulin G-1 monoclonal anti-CD20 antibody, has proven successful in treating hematologic malignancies. B cells play a vital role in immune functions, including cytokine and immunoglobulin production, and antigen presentation.^10,11^ Gastrointestinal mucosal immunity, involving the innate immune system, T cells, B cells, and associated cytokines, is crucial for balancing proinflammatory and anti-inflammatory stimuli.

**Figure 2.**
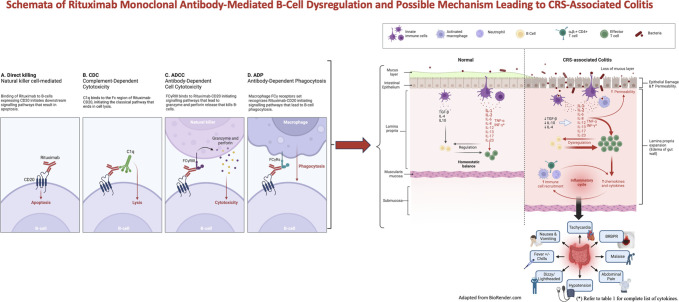
Schemata of proposed mechanisms underlying B-cell depletion and dysregulation mediated by rituximab monoclonal antibody, leading to subsequent CRS-associated colitis. *Refer to Table 1 for a complete list of cytokines. CRS, cytokine release syndrome.

Although the precise mechanism behind RTX-associated colitis remains unclear, the development of de novo colitis after the initiation of RTX suggests the protective role of B cells in maintaining healthy intestinal mucosa. One potential mechanism involves B-cell depletion, which disrupts intestinal mucosal immunoregulation, leading to increased infiltration of T lymphocytes.^12^ This results in a decrease in interleukin (IL)-4, IL-10, and transforming growth factor β and an increase in CD4^+^ T cells releasing inflammatory cytokines such as IL-1, IL-6, IL-17, tumor necrosis factor-α, and interferon-γ. causing cytokine release syndrome (CRS). The intricate interplay and the key cytokines associated with CRS-related colitis are detailed in Table 1, and the schematics are depicted in Figure 2. It is noteworthy that the interactions are complex, and the listed cytokines may have overlapping roles in mechanisms.

**Table 1.** The potential pathways and key cytokines involved in CRS-associated colitis

	Mechanisms	Key cytokines involved
1	Epithelial damage (disruption of barrier function), immune cell infiltration, activation, mucosal damage, vasodilation, increased permeability, coagulation abnormalities, and platelet dysfunction	IL-1, IL-2, IL-6, IL-8, IL-17, TNF-α, IFN-γ, GM-CSF, MCP-1, MIP-1α, MIP-1β, IP-10, and RANTES
2	Release of proinflammatory mediators	Prostaglandins, leukotrienes, TNF-α, IL-1, IL-6, IL-8, MCP-1, MIP-1α, and MIP-1β
3	Direct cytotoxic effects on gut lining	Perforin and granzyme B (in severe cases)
4	Activation of complement system	Complement proteins, C3a, and C5a
5	Anti-inflammatory and immune-modulatory response	IL-4, IL-10, and TGF-β

CRS, cytokine release syndrome; IFN, interferon; IL, interleukin; GM-CSF, granulocyte–macrophage colony-stimulating factor; MCP-1, monocyte chemoattractant protein-1; MIP-1α, macrophage inflammatory protein-1 alpha; MIP-1β, macrophage inflammatory protein-1 beta; IP-10, interferon-gamma–induced protein 10; RANTES, regulated on activation, normal T cell expressed and secreted; TGF, transforming growth factor; TNF, tumor necrosis factor.

CRS-associated colitis typically occurs approximately 90 minutes after infusion, disrupting local homeostasis of the intestinal barrier, triggering inflammation, and increasing membrane permeability in the intestinal tract.^12^ Cytokine imbalances may cause systemic vasodilation, resulting in severe hypotension. In severe cases of CRS after RTX, a 5- to 10-fold increase in liver enzymes, elevation of D-dimer, lactate dehydrogenase, and prolongation of prothrombin time are also commonly observed.^13^ It is unclear why our patient did not experience any side effects or colitis during her first dose of RTX; however, it is noteworthy that colitis manifested during the administration of the fourth dose, suggesting a possible role of cumulative damage to the colonic mucosa in this development or possible colonic ischemia because of hypotension.

RTX has been associated with diverse types of colitis, with a total of 35 reported cases, including ulcerative colitis (UC), Crohn's disease, microscopic colitis (MC), and ileocolitis. Among these, 13 patients developed RTX-induced UC,^3,14–16^ 11 had RTX-induced Crohn's disease,^15,17–19^ 9 had RTX-induced MC, and 2 had ileocolitis.^7,20^ A case report also suggested a link between colitis and Toro virus infection after RTX treatment, although our patient's stool polymerase chain reaction (PCR) panel was negative. Given our patient's rapid improvement within 24 hours, the colonoscopy was deferred. Although the specific type of colitis was not confirmed, the swift recovery suggests a possibility of inflammatory or ischemic colitis.

The reason why different types of RTX-induced colitis (UC, Crohn's disease, MC, and ileocolitis) occur in distinct patients remains unclear. One potential mechanism may involve the varying suppression of regulatory T cells and activation of Th1 and Th17 induced by B-cell depletion after RTX, leading to differences in the degree of inflammation and affected regions in the intestinal mucosa. Understanding this mechanism could shed light on the pathogenesis of colitis.

In summary, RTX therapy is widely used in hematologic malignancies and autoimmune diseases, but colitis is an underappreciated complication. B cells play dual roles, inflammatory and anti-inflammatory roles, and an imbalance may result in colitis. RTX-induced colitis should be considered a differential diagnosis for patients experiencing bloody or watery stool after RTX treatment. Moreover, gathering cases is essential to establish diagnostic criteria and determine the optimal treatment for RTX-induced colitis.

## DISCLOSURES

Author contributions: F. Vikash and D. Wan devised the idea to publish this rare case and wrote a manuscript draft, revision, and generating the schemata. S. Vikash helped in writing manuscript draft, revision, and revising the schemata. M. Mintz helped with image editing and finalizing it. S. Elshafey and D. Kim helped with the manuscript revision. D. Wan is the attending gastroenterologist and guarantor of the article.

Financial disclosure: None to report.

Previous presentation: This case was presented as a poster at the American College of Gastroenterology Annual Meeting 2023; Vancouver, Canada.

Informed consent was obtained for this case report.
